# Supplementary figures and images for: A retrospective investigation of the population structure and geospatial distribution of Salmonella Paratyphi A in Kathmandu, Nepal
Source: PLoS Negl Trop Dis. 2024 Jun 18;18(6):e0011864. doi: 10.1371/journal.pntd.0011864 (PMC11216570; doi:10.1371/journal.pntd.0011864)

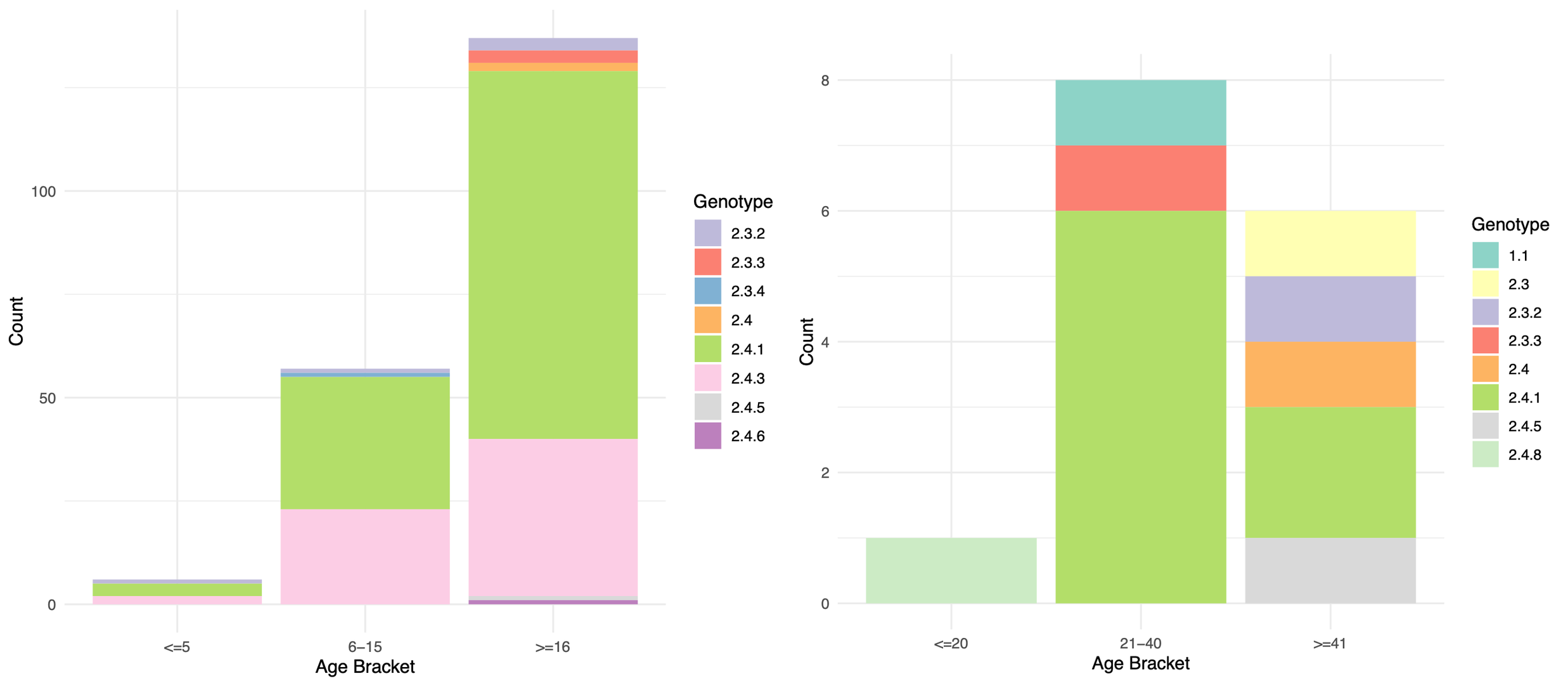

Supplement: S1 Fig — Bar charts show counts of patients in each age bracket. (TIF) [file pntd.0011864.s003.tif]

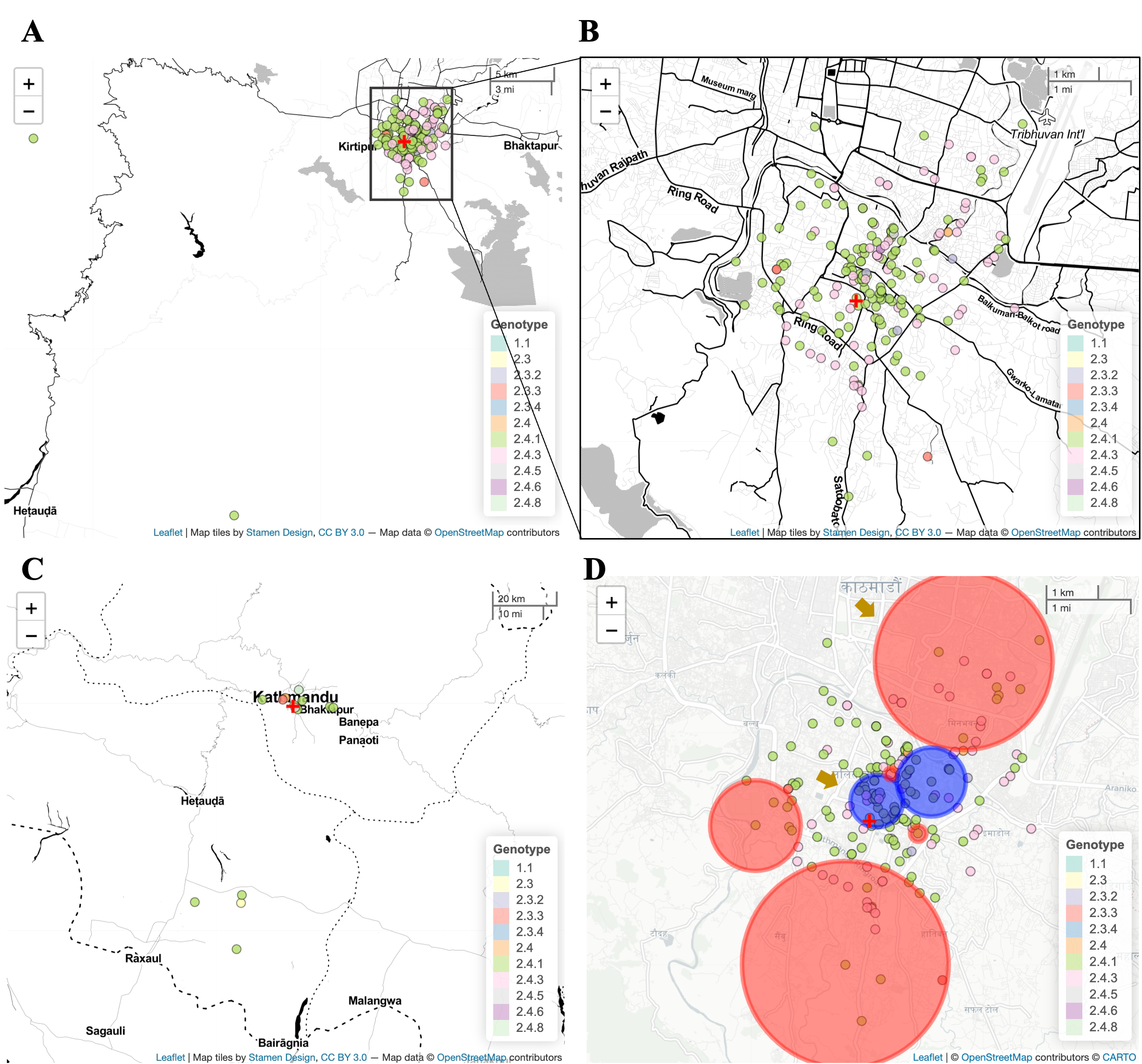

Supplement: S2 Fig — (A) Genotypes of acute S. Paratyphi A organisms with (B) a zoomed in inset of the majority of cases. (C) Genotypes of carrier S. Paratyphi A organisms. (D) Spatiotemporal clustering of S. Paratyphi A with red circles showing high-rate clusters and the blue circles indicating low-rate clusters (statistically significant clusters, p<0.05, are indicated with yellow arrows). Red cross indicates the Patan Hospital. Map bases are available from OpenStreetMap and are under the Open Database License (https://www.openstreetmap.org/copyright). (TIF) [file pntd.0011864.s004.tif]

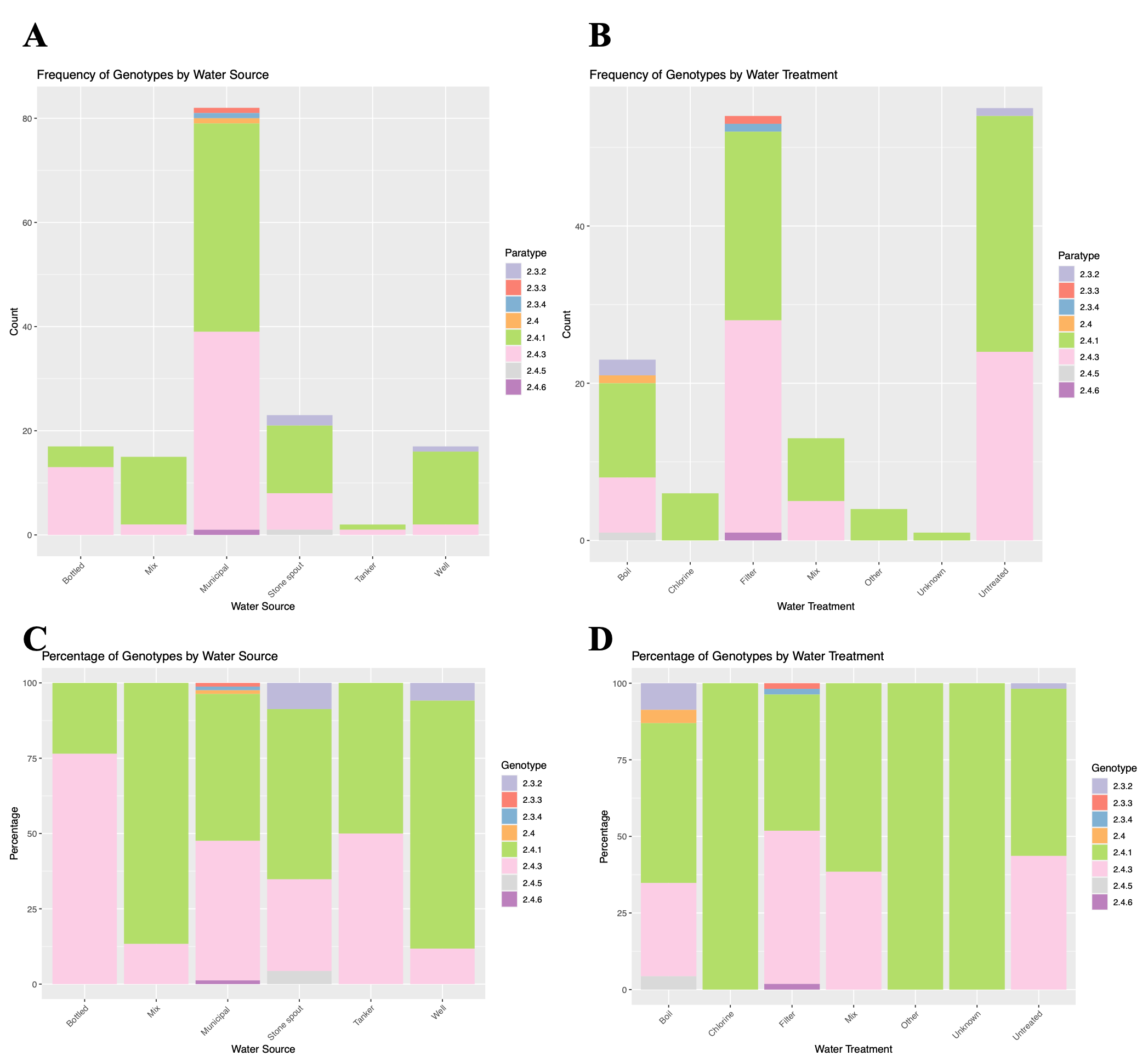

Supplement: S3 Fig — Stacked bar plots showing the (A-B) count and (C-D) percentages of each genotype for each associated water source (A-C) and water treatment (B-D). (TIF) [file pntd.0011864.s005.tif]
